# Prevalence, clinical characteristics, and predictors of sleep disordered breathing in hospitalized heart failure patients

**DOI:** 10.1002/clc.23925

**Published:** 2022-09-30

**Authors:** Boping Huang, Yan Huang, Mei Zhai, Qiong Zhou, Shiming Ji, Huihui Liu, Xiaofeng Zhuang, Yuhui Zhang, Jian Zhang

**Affiliations:** ^1^ Heart Failure Center State Key Laboratory of Cardiovascular Disease, Fuwai Hospital, National Center for Cardiovascular Diseases, Chinese Academy of Medical Sciences and Peking Union Medical College (CAMS & PUMC) Beijing China

**Keywords:** central sleep apnea, heart failure, obstructive sleep apnea, predictor

## Abstract

**Background:**

Heart failure (HF) is often comorbid with sleep disordered breathing (SDB). This prospective study investigated the prevalence, clinical characteristics, and predictors of SDB in hospitalized HF patients.

**Methods:**

Sleep studies were performed on hospitalized HF patients from January 2015 to February 2019. SDB was categorized as no/mild SDB, obstructive sleep apnea (OSA), and central sleep apnea (CSA).

**Results:**

The study included 1069 hospitalized HF patients. The prevalence rates of OSA and CSA were 16.6% and 36.9%, respectively. Patients with OSA or CSA were more likely to be male and have a higher body mass index (BMI) and more comorbidities. Multivariate logistic regression analysis showed that male sex (odds ratio [OR] = 1.803, 95% confidence interval  [CI] = 1.099–2.958), BMI (per 5 kg/m^2^ increase: OR = 2.270, 95% CI = 1.852–2.783), hypertension (OR = 2.719, 95% CI = 1.817–4.070), diabetes (OR = 1.477, 95% CI = 1.020–2.139), and left ventricular ejection fraction (LVEF) (per 5% increase, OR = 1.126, 95% CI = 1.053–1.204) were independent predictors of OSA. Male sex (OR = 1.699, 95% CI = 1.085–1.271), age (per 10 years, OR = 1.235, 95% CI = 1.118‐1.363), heart rate (per 10 bpm, OR = 1.174, 95% CI = 1.099–2.958), LVEF (per 5% increase, OR = 0.882, 95% CI = 0.835–0.932), NT‐proBNP (lnNT‐proBNP, OR = 1.234, 95% CI = 1.089–1.398) and hypocapnia (OR = 1.455, 95% CI = 1.105–1.915) were independent predictors of CSA. The areas under the receiver operating characteristic curves were 0.794 (95% CI = 0.758–0.830) and 0.673 (95% CI = 0.640–0.706), respectively.

**Conclusions:**

More than half of hospitalized HF patients had OSA or CSA, and CSA was the predominant type. OSA and CSA predictors differ. The clinical characteristics of HF patients can help make preliminary predictions for SDB patients.

## 1 INTRODUCTION

Sleep disordered breathing (SDB) is a highly prevalent comorbidity of heart failure (HF).^1^, ^2^, ^3^, ^4^, ^5^ Due to the pathophysiology of HF, decreased cardiac function, increased pulmonary venous pressure, and increased fluid load often contribute to the occurrence and aggravation of SDB.^6^ SDB, through mechanisms such as nocturnal hypoxemia, the sympathetic nervous system, renin‐angiotensin‐aldosterone system activation, and chronic inflammation, worsen HF.^7^, ^8^, ^9^ SDB increases the risk of death and rehospitalization, which are independently associated with a poor prognosis for HF.^10^, ^11^, ^12^

SDB mainly includes obstructive sleep apnea (OSA) and central sleep apnea (CSA). Positive airway pressure therapy has been shown to attenuate sleep apnea and daytime sleepiness and improve quality of life and left ventricular ejection fraction (LVEF)^13^, ^14^; whether it effectively improves the prognosis of HF is still controversial.^15^ The SERVE‐HF study found that adaptive servo‐ventilation therapy did not significantly affect the prognosis of HF but increased the risk of cardiovascular and all‐cause mortality.^16^ In these patients, implantable phrenic nerve stimulation may provide new possibilities for symptomatic relief.^17^ Considering the frequent co‐occurrence of SDB in HF and its adverse prognosis, early diagnosis and treatment of SDB may be beneficial. Subjective daytime sleepiness symptoms are often absent in HF patients with SDB and are therefore often missed. Thus, use of the clinical characteristics of patients with HF can provide clues for the diagnosis of SDB, potentially increasing the detection rate of SDB.

Prospective population studies have shown that SDB affects 46%–76% of patients with stable HF^1^, ^18^; however, there are limited data on hospitalized HF.^4^, ^5^ Therefore, we aimed to investigate the prevalence, clinical characteristics, and predictors of SDB in hospitalized HF patients through clinical information, including demographics, echocardiography, and plasma markers, in a large sample population.

## 2 METHODS

### 2.1 Patients

This prospective study included patients with HF hospitalized in the HF Center of Fuwai Hospital between January 2015 and February 2019. HF was defined according to at least one symptom and one sign of congestive HF (dyspnea or fatigue, volume overload, chest X‐ray), elevated NT‐proBNP level, and echocardiography. The inclusion criteria were acute new‐onset HF and chronic decompensated HF; NT‐proBNP >300 pg/ml; no limitation on LVEF; and New York Heart Association (NYHA) Classes Ⅱ–Ⅳ. We excluded patients younger than 18 years; history of malignant tumor; chronic obstructive pulmonary disease; previous diagnosis of SDB; long‐term oxygen or any positive pressure ventilation therapy. Within 48 h of admission, an echocardiogram was performed, and the LVEF was measured by using the Simpson method. HF is divided into HFpEF (HF with reduced ejection fraction), HFmrEF (HF with mid‐range ejection fraction), and HFrEF (HF with reduced ejection fraction) according to LVEF ≤40%, 40%–49%, ≥50%. Hypocapnia was defined as a partial pressure of arterial carbon dioxide ≤38 mmHg. The protocol was approved by the Fuwai Hospital Research Ethics Board. The study conformed to the Declaration of Helsinki, and all patients signed informed consent before enrollment.

### 2.2 Sleep studies

Patients received overnight sleep monitoring using Apnealink Plus (Resmed) during hospitalization. The device records and measures the patient's nasal airflow, snoring, and thoracoabdominal motion. Oxygen saturation and pulse rate were monitored by finger pulse oximetry continuously. Patients were assessed for nighttime sleep monitoring within 48 h of admission or during hospitalization. During sleep monitoring, the patient's vital signs were stable, without hypotension (systolic blood pressure <90 mmHg), without assisted oxygen inhalation, and with no need for invasive or noninvasive ventilator‐assisted ventilation. Experienced night shift nurses regularly checked the placement of the monitoring device and made necessary adjustments to ensure the integrity of the recording. Only periods with sufficient airflow and oxygen saturation signals at the same time were regarded as an effective recording. We considered only sleep monitoring with at least 4 h of adequate recording time as described previously.^19^ Based on the American Academy of Sleep Medicine,^20^ apnea and hypopnea were scored as a ≥90% and ≥30% decrease in breathing amplitude, respectively, for ≥10 s. At the same time, hypopnea‐related decreases were ≥3% oxygen desaturation. The apnea‐hypopnea index (AHI) was determined using apnea plus hypopnea events per hour of sleep. SDB was classified as no, mild, moderate, and severe using AHI cutoffs of 5, 15, and 30. We characterized moderate‐to‐severe SDB as an AHI of 15 or above. If the central apnea or hypopnea episodes in patients with moderate to severe SDB were higher than 50%, they were categorized as CSA; otherwise, they were classified as OSA. The two sleep physicians were blinded to the clinical status of the participants.

### 2.3 Statistical analysis

Continuous and categorical variables are expressed as the mean ± *SD* and counts and proportions (%), respectively. Variables with skewed distributions were transformed using the natural logarithm. Continuous variables were compared using the Kruskal‒Wallis test. Associations between clinical variables and the predictors of SDB were analyzed using binary logistic regression models. The relative risk levels were expressed by odds ratios (ORs) and 95% confidence intervals (CIs). Variables that were considered clinically relevant or statistically significant in univariate analysis were included in the multivariate analysis. The multivariate logistic regression model was selected based on stepwise forward selection using a likelihood ratio test with a significance level of 0.1 for exclusion and 0.05 for re‐entry. Variables for inclusion were carefully chosen based on the number of available events to ensure the simplicity of the final model. Candidate predictors were all variables in the baseline table except sleep monitoring variables. Discrimination and calibration of predictive models were assessed with the area under the receiver operating characteristic (ROC) curve and Hosmer‒Lemeshow goodness‐of‐fit tests. Analyses were performed in SPSS version 25.0 and R version 3.4.6.

## 3 RESULTS

### 3.1 Patient characteristics

The study ultimately included 1069 patients for analysis (Figure S1). The average age and body mass index (BMI) were 55.7 ± 15.0 years and 24.9 ± 4.6 kg/m^2^, respectively. There were 656 (62.2%), 162 (15.2%), and 242 (22.6%) patients with HFpEF, HFmrEF, and HFrEF, respectively. The overall prevalence of moderate‐to‐severe SDB was 53.4% (571), including 177 (16.6%) OSA and 394 (36.9%) CSA patients. Compared with nmSDB, the prevalence of coronary heart disease, hypertension, and diabetes was higher in OSA and CSA. Patients with OSA were more likely to be male and have a higher BMI, and higher systolic and diastolic blood pressure levels (Table 1). Patients with CSA were more likely to be male and have NYHA Class IV, higher NT‐proBNP levels, and lower LVEF. OSA and CSA had significantly longer periods of <90% oxygen saturation and lower mean and minimum oxygen saturations than nmSDB.

**Table 1:** Clinical characteristics of hospitalized heart failure patients

	Total (*n* = 1069)	nmSDB (*n* = 498)	OSA (*n* = 177)	CSA (*n* = 394)	*p* value
Age (years)	55.7 (15.0)	54.8 (15.5)	56.5 (15.5)	56.6 (14.1)	.147
Male, *n* (%)	813 (76.1)	338 (67.9)	150 (84.7)	325 (82.5)	<.001
BMI (kg/m^2^)	24.9 (4.6)	23.8 (4.1)	28.5 (5.2)	24.7 (4.0)	<.001
Smoking, *n* (%)	211 (19.7)	81 (16.3)	39 (22.0)	91 (23.1)	.028
Drinking, *n* (%)	387 (36.2)	161 (32.3)	64 (36.2)	162 (41.1)	.025
Coronary artery disease, *n* (%)	413 (38.6)	169 (33.9)	81 (45.8)	163 (41.4)	.008
Hypertension, *n* (%)	503 (47.1)	186 (37.3)	136 (76.8)	181 (45.9)	<.001
Diabetes, *n* (%)	312 (29.2)	118 (23.7)	77 (43.5)	117 (29.7)	<.001
Atrial fibrillation, *n* (%)	393 (36.8)	193 (38.8)	62 (35.0)	138 (35.0)	.451
Heart rate (bpm)	78.3 (17.1)	75.5 (16.5)	79.8 (17.0)	81.1 (17.3)	<.001
Systolic BP (mmHg)	118.2 (20.1)	116.1 (19.8)	128.8 (20.2)	116.1 (19.0)	<.001
Diastolic BP (mmHg)	73.2 (13.3)	71.2 (12.6)	78.8 (13.2)	73.4 (13.4)	<.001
NYHA, *n* (%)					.004
Ⅱ	174 (16.3)	83 (16.7)	39 (22.0)	52 (13.2)	
Ⅲ	569 (53.2)	270 (54.2)	99 (55.9)	200 (50.8)	
Ⅳ	326 (30.5)	145 (29.1)	39 (22.0)	142 (36.0)	
Hemoglobin (g/L)	140.9 (22.4)	139.2 (23.0)	144.3 (23.4)	141.4 (21.1)	.029
eGFR (ml/min/1.73 m^2^)	74.3 (25.5)	75.4 (26.2)	74.1 (27.0)	72.9 (23.7)	.351
LDL‐C (mmol/L)	2.4 [1.8, 3.0]	2.4 [1.8, 2.9]	2.5 [1.9, 3.2]	2.3 [1.8, 3.1]	.111
Hypocapnia, *n* (%)	632 (59.1)	280 (56.2)	89 (50.3)	263 (66.8)	<.001
NT‐proBNP (pg/ml)	2837 [1279, 6785]	2509 [1169, 6055]	1934 [899, 5019]	3853 [1834, 8323]	<.001
LVEF (%)	37.7 (13.9)	39.2 (14.6)	40.6 (13.7)	34.6 (12.4)	<.001
<40%	665 (62.2)	288 (57.8)	97 (54.8)	280 (71.1)	<.001
40%–49%	162 (15.2)	80 (16.1)	29 (16.4)	53 (13.5)	
≥50%	242 (22.6)	130 (26.1)	51 (28.8)	61 (15.5)	
ACEIs/ARBs, *n* (%)	529 (49.5)	236 (47.4)	91 (51.4)	202 (51.3)	.441
Beta‐blocker, *n* (%)	737 (68.9)	343 (68.9)	112 (63.3)	282 (71.6)	.140
Spironolactone, *n* (%)	689 (64.5)	321 (64.5)	92 (52.0)	276 (70.1)	<.001
Digoxin, *n* (%)	274 (25.6)	125 (25.1)	39 (22.0)	110 (27.9)	.308
Diuretic, *n* (%)	793 (74.2)	366 (73.5)	121 (68.4)	306 (77.7)	.056
AHI (events/h)	19.8 (15.3)	6.9 (4.4)	31.6 (11.7)	30.8 (12.5)	<.001
ODI (events/h)	23.3 (15.2)	11.6 (7.1)	36.2 (12.8)	32.4 (12.7)	<.001
Mean oxygen saturation (%)	93.7 (3.2)	94.3 (3.4)	92.4 (3.6)	93.6 (2.5)	<.001
Minimum oxygen saturation (%)	82.3 (8.5)	84.8 (8.8)	77.8 (8.8)	81.2 (6.7)	<.001
Time <90% oxygen saturation (min)	16.7 (23.1)	11.7 (22.6)	26.6 (24.9)	18.6 (21.2)	<.001

Abbreviations: ACEI, angiotensin‐converting enzyme inhibitor; AHI, apnea‐hypopnea index; ARB, angiotensin receptor blocker; BMI, body mass index; BP, blood pressure; CSA, central sleep apnea; eGFR, estimated glomerular filtration rate; LDL‐C, low‐density lipoprotein cholesterol; LVEF, left ventricular ejection fraction; nmSDB, no or mild sleep disordered breathing; NT‐proBNP, N‐terminal pro‐brain natriuretic peptide; NYHA, New York Heart Association; ODI, oxygen desaturation index; OSA, obstructive sleep apnea.

### 3.2 Prevalence characteristics of SDB

**Figure 1:**
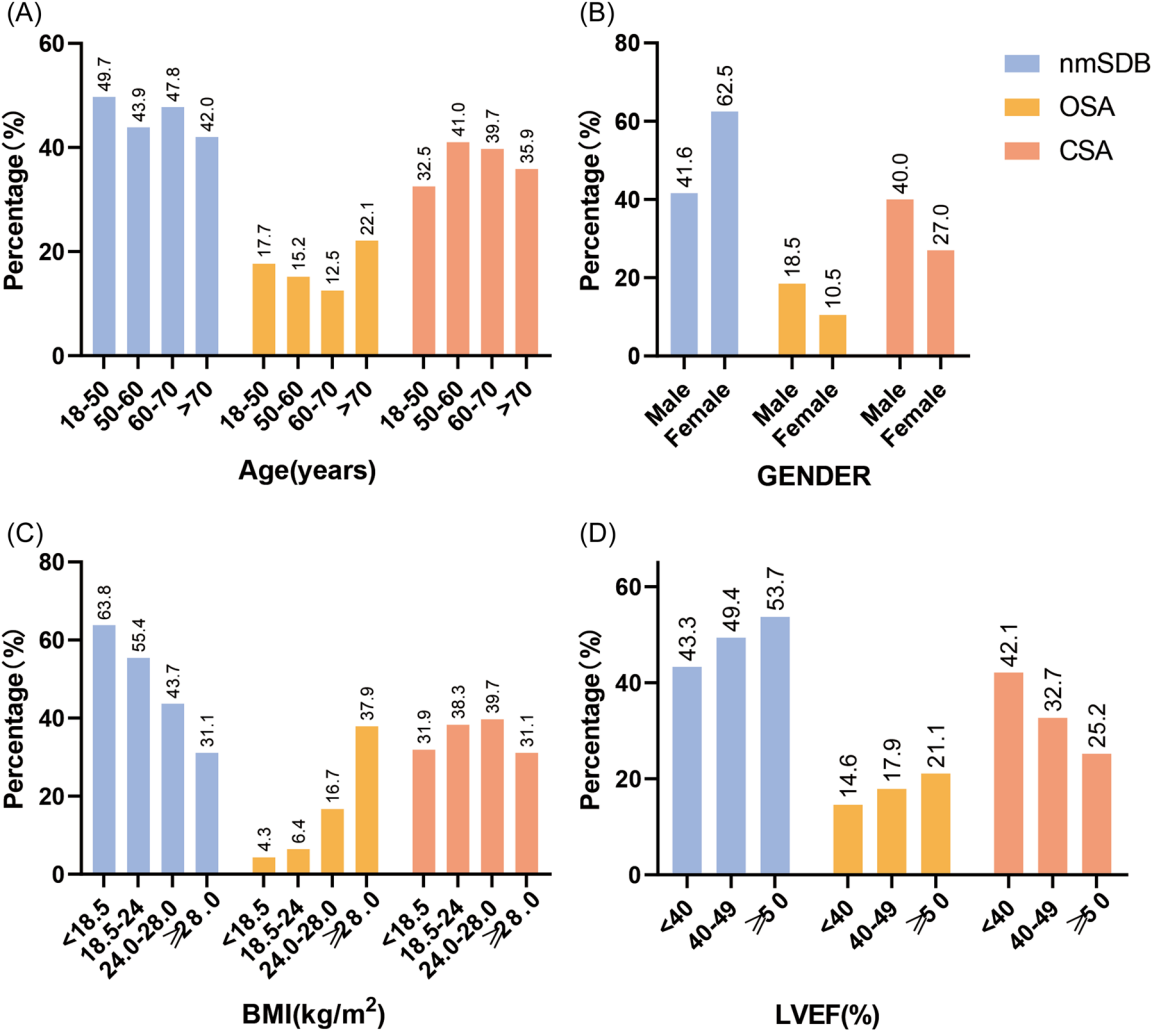
Prevalence characteristics of SDB classification. (A–D) represent the prevalence of SDB in different sex (A), age (B), BMI (C), and LVEF (D) groups of patients. BMI, body mass index; CSA, central sleep apnea; LVEF, left ventricular ejection fraction; nmSDB, no or mild sleep‐disordered breathing; OSA, obstructive sleep apnea

The prevalence of SDB varied between males and females. Compared with females, males had a higher prevalence of OSA (18.5% vs. 10.5%) and CSA (40.0% vs. 27.0%) (Figure 1). The prevalence of OSA and CSA did not increase with age. With an increase in BMI, the prevalence of OSA showed an upward trend. There was a difference in moderate‐to‐severe SDB prevalence among patients with different LVEF levels, and the prevalence of OSA increased with LVEF. With the increase in LVEF, CSA prevalence showed a downward trend. The prevalence rates of moderate‐to‐severe SDB in HFrEF, HFmrEF, and HFpEF were 56.7%, 50.6%, and 46.3%, respectively.

### 3.3 Predictors of OSA and CSA

**Figure 2:**
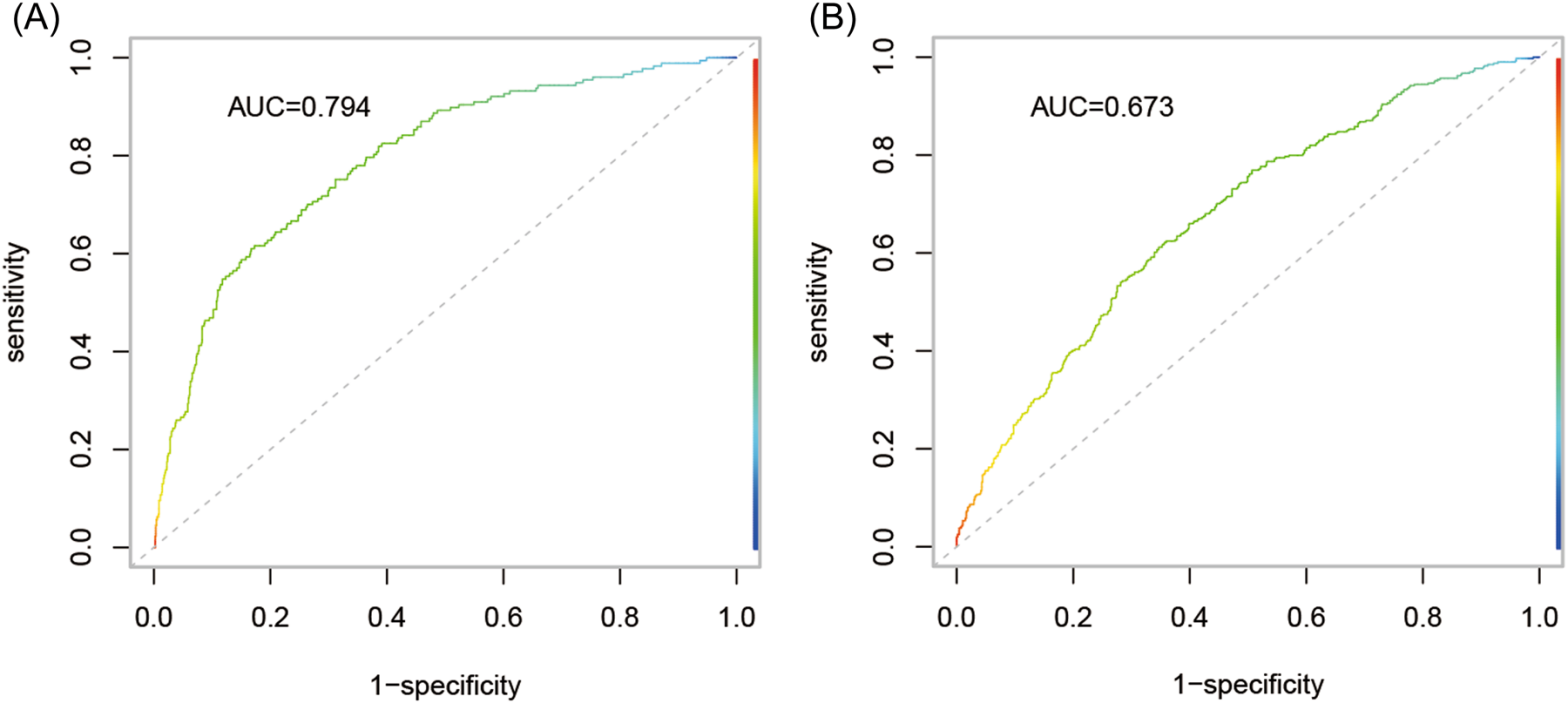
Area under the ROC curve of OSA (A) and CSA (B) prediction models. AUC, area under the ROC curve; CSA, central sleep apnea; OSA, obstructive sleep apnea; ROC, receiver operating characteristic

Univariate logistic regression found that OSA was related to male sex, BMI, coronary heart disease, hypertension, diabetes, hemoglobin level, NT‐proBNP level, LVEF, and hypocapnia (all *p* < .10). The multivariate logistic regression analysis revealed that in hospitalized HF patients, male sex (OR = 1.803, 95% CI = 1.099–2.958, *p* = .020), BMI (per 5 kg/m^2^ increase: OR = 2.270, 95% CI = 1.852–2.783, *p* < .001), hypertension (OR = 2.719, 95% CI = 1.817–4.070, *p* = .001), diabetes (OR = 1.477, 95% CI = 1.020–2.139, *p* = .039), and LVEF (per 5% increase, OR = 1.126, 95% CI = 1.053–1.204, *p* = .001) were independently associated with OSA (Table 2). The area under the ROC curve (0.794, 95% CI = 0.758–0.830, *p* < .001) and Hosmer‒Lemeshow goodness‐of‐fit test (*p* = .290) demonstrated good discrimination and good calibration capability of the OSA prediction model (Figure 2).

**Table 2:** Correlation between clinical variables and obstructive sleep apnea

	Univariate logistic regression			Multivariate logistic regression		
	OR	95% CI	*p* value	OR	95% CI	*p* value
Age (per 10 years)	1.042	0.935–1.162	.453	–	–	–
Male	1.919	1.240–2.969	.003	1.803	1.099–2.958	.020
BMI (per 5 kg/m^2^)	2.653	2.190–3.215	<.001	2.270	1.852–2.783	<.001
Coronary artery disease	1.423	1.028–1.970	.034	–	–	–
Hypertension	4.745	3.265–6.896	<.001	2.719	1.817–4.070	.001
Diabetes	2.153	1.544–3.002	<.001	1.477	1.020–2.139	.039
Atrial fibrillation	0.914	0.652–1.280	.600	–	–	–
Heart rate (per 10 bpm)	1.065	0.971–1.168	.183	–	–	–
Hemoglobin	1.008	1.001–1.016	.027	–	–	–
lnNT‐proBNP	0.747	0.643–0.868	<.001	–	–	–
LVEF (per 5%)	1.089	1.030–1.153	.003	1.126	1.053–1.204	.001
Hypocapnia	0.650	0.470–0.899	.009	–	–	–

Abbreviations: ACEI, angiotensin‐converting enzyme inhibitor; ARB, angiotensin receptor blocker; BMI, body mass index; CI, confidence interval; LVEF, left ventricular ejection fraction; NT‐proBNP, N‐terminal pro‐brain natriuretic peptide; OR, odds ratio.

The univariate logistic regression of CSA is shown in Table 3. The multivariate logistic regression analysis showed that male sex (OR = 1.699, 95% CI = 1.220–2.366, *p* = .002), age (per 10 years: OR = 1.235, 95% CI = 1.118–1.363, *p* < .001), heart rate (per 10 bpm, OR = 1.174, 95% CI = 1.085–1.271, *p* < .001), NT‐proBNP level (lnNT‐proBNP, OR = 1.234, 95% CI = 1.089–1.398, *p* = .001), LVEF (per 5%, OR = 0.882, 95% CI = 0.835–0.932, *p* < .001), and hypocapnia (OR = 1.455, 95% CI = 1.105–1.915, *p* = .007) were independently related to CSA (Table 3). The CSA prediction model had an area under the ROC curve of 0.673 (95% CI = 0.640–0.706) with good discrimination (Figure 2) and good calibration (*p* = .594 for the Hosmer‒Lemeshow goodness‐of‐fit test).

**Table 3:** Correlation between clinical variables and central sleep apnea

	Univariate logistic regression			Multivariate logistic regression		
	OR	95% CI	*p* value	OR	95% CI	*p* value
Age (per 10 years)	1.063	0.978–1.156	.148	1.235	1.118–1.363	<.001
Male	1.805	1.324–2.460	<.001	1.699	1.220–2.366	.002
BMI (per 5 kg/m^2^)	0.916	0.799–1.051	.212	–	–	–
Coronary artery disease	1.200	0.930–1.547	.161	–	–	–
Hypertension	0.932	0.726–1.195	.577	–	–	–
Diabetes	1.040	0.791–1.366	.780	–	–	–
Atrial fibrillation	0.888	0.685–1.150	.368	–	–	–
Heart rate (per 10 bpm)	1.166	1.083–1.255	<.001	1.174	1.085–1.271	<.001
Hemoglobin	1.002	0.996–1.007	.539	–	–	–
lnNT‐proBNP	1.393	1.240–1.563	<.001	1.234	1.089–1.398	.001
LVEF (per 5%)	0.873	0.832–0.916	<.001	0.882	0.835–0.932	<.001
Hypocapnia	1.665	1.285–2.156	<.001	1.455	1.105–1.915	.007

Abbreviations: ACEI, angiotensin‐converting enzyme inhibitor; ARB, angiotensin receptor blocker; BMI, body mass index; CI, confidence interval; LVEF, left ventricular ejection fraction; NT‐proBNP, N‐terminal pro‐brain natriuretic peptide; OR, odds ratio.

## 4 DISCUSSION

This single‐center large‐sample prospective cohort study reported the prevalence and clinical characteristics of SDB in consecutive hospitalized HF patients and identified independent predictors of SDB from a series of clinical variables. SDB is very common among hospitalized HF patients. The prevalence of moderate‐to‐severe SDB was 53.4%, more than one‐third of these patients had CSA (36.9%), and 16.6% had OSA. The clinical features of OSA and CSA vary. OSA and CSA both had a higher proportion of male sex, higher BMI, higher heart rate, and more comorbidities. However, OSA had higher blood pressure levels, and CSA had higher NT‐proBNP levels and lower LVEF. Male sex, BMI, hypertension, diabetes, and LVEF are independent predictors of OSA. Conversely, age, male sex, heart rate, NT‐proBNP levels, LVEF, and hypocapnia are independent predictors of CSA.

The prevalence of SDB is higher among hospitalized HF patients than among stable chronic HF patients.^8^ Prospective population studies have demonstrated that the bulk of moderate‐to‐severe SDB in stable chronic HF was 46%.^1^ However, Khayat et al. reported that among 395 hospitalized patients with decompensated HF, with AHI ≥ 15 events/h as the diagnostic criteria, the prevalence of SDB was approximately 75%, with 57% of these patients having predominantly OSA, and 18% demonstrating CSA.^3^ In a study including 1117 patients with acute HF, the prevalence of OSA and CSA was 47.0% and 30.8%, respectively.^10^

Some reasons could explain the different prevalence rates of SDB. First, we enrolled hospitalized HF patients for sleep monitoring, regardless of whether they had nocturnal hypoxemia or daytime drowsiness. Second, the lower BMI level in this study may have resulted in a lower proportion of patients with OSA compared to the previous two studies. Obesity is considered to be one of the main factors contributing to OSA, as it can affect the anatomy of the airway, increase the risk of glossoptosis, and reduce lung capacity. Third, our study found that the prevalence of SDB decreased with increasing LVEF. Patients with HFrEF mainly had CSA, while the proportion of OSA increased in HFpEF, suggesting that the mechanism of SDB in patients with different ejection fractions may be different. Furthermore, when patients with HF lie down at night, fluid from the lower limbs transfers to the lungs and pharynx, increasing the risk of upper airway stenosis, reducing hyperventilation and arterial carbon dioxide pressure, and exacerbating OSA and CSA.^21^, ^22^

Correlations between SDB and age, sex, BMI, and LVEF were found in stable chronic HF.^1^, ^22^ Our study, through multivariate logistic regression analysis, found that male sex, BMI, hypertension, diabetes, and LVEF were independent correlated factors of OSA, indicating a close link between OSA and metabolic syndrome.^23^ Age, male sex, heart rate, LVEF, NT‐proBNP levels, and hypocapnia are independent correlated factors of CSA in hospitalized patients with HF, suggesting that CSA is more likely to be the indicator and result of the severity of HF. Hypocapnia is a crucial determinant factor of CSA.^24^ Similar to previous studies, our study also found that male sex, older age, and hypocapnia were predictors of CSA.^25^ Moreover, our study found that the differences in clinical predictors of OSA and CSA may be due to different mechanisms of OSA and CSA.

The area under the ROC curve and the Hosmer‒Lemeshow goodness‐of‐fit test indicate that the prediction models of OSA and CSA have good discrimination and calibration. The clinical characteristics of hospitalized patients with HF can provide insights for the risk assessment of SDB, where discovery may provide clues for clinical work. OSA and CSA are underdiagnosed conditions. Patients with systolic HF show less subjective daytime sleepiness. Such scales as the STOP‐BANG, Epworth Sleepiness Scale, and Berlin questionnaires may not be suitable for assessing the risk of patients with HF and SDB, regardless of the presence or absence of SDB.^26^ Our research demonstrates that clinical data, including demographic characteristics, echocardiography, and plasma markers, provide specific values for the risk assessment of SDB. The design of a risk assessment system including clinical data may be helpful for the early detection and diagnosis of SDB.

However, assessing clinical characteristics was only the first step in SDB screening. Considering that SDB is highly prevalent among hospitalized HF patients, relevant sleep monitoring should be widely used in the clinical diagnosis of SDB, especially for high‐risk patients. Laboratory polysomnography is the gold standard for diagnosing SDB, but the complexity of polysomnography is not completely suitable for hospitalized HF patients. Portable sleep monitoring showed robust agreement with polysomnography, which can be used to accurately and efficiently diagnose SDB.^27^ With the advantages of simple operation, portable sleep monitoring has been widely used in cardiovascular clinical and research work. The oxygen desaturation index in patients with HF correlated with the AHI,^28^ suggesting that simple blood oxygen saturation monitoring may help screen for SDB.

This study has the following advantages. First, this is an unselected, consecutively enrolled large‐sample prospective HF cohort study. Second, the study population included patients with OSA and CSA and predicted using common clinical variables. Third, this cohort includes HF with preserved, mid‐range, and reduced ejection fractions. This study also has some shortcomings. We used portable sleep monitoring, not polysomnography, to evaluate SDB. Portable sleep monitoring does not involve brain electrodes. It cannot analyze wake or sleep status through brain electrical activity, so it is impossible to determine the actual sleep time at night. We can only estimate the AHI by evaluating the overall recording time. The actual sleep time at night is shorter than the recording time, so the AHI value calculated by portable sleep monitoring may be lower than the actual value.^20^ On the other hand, because the monitoring equipment used in this study has only chest motion signal detection, misclassification may exist. Finally, considering the different pathophysiological mechanisms of OSA and CSA, future studies should further evaluate the impact of different clinical phenotypes of SDB on treatment choices in hospitalized patients with HF.

## 5 CONCLUSIONS

This prospective cohort study reported the prevalence and clinical characteristics of SDB in hospitalized HF patients, identifying independent predictors of SDB by common clinical variables. CSA is the predominant type of hospitalized HF. More than half of the patients had OSA or CSA. OSA patients were more likely to be male and obese and have hypertension, diabetes, and HFpEF. CSA patients were more likely to be older and male and have hypocapnia, high heart rate, higher NT‐proBNP levels, and HFrEF. The patient's clinical characteristics, including demographics, echocardiography, and cardiac biomarkers, were used to help make preliminary predictions for patients with SDB.

## CONFLICTS OF INTEREST

The authors declare no conflicts of interest.

## Supporting information

Supporting information.

## Data Availability

Data is not available due to the restrictions of this research. The authors confirm that material supporting the findings of this study can be found in the article and its supplementary materials.
