# Supplementary material for: Prevalence, clinical characteristics, and predictors of sleep disordered breathing in hospitalized heart failure patients
Source: Clin Cardiol. 2022 Sep 30;45(12):1311–8. doi: 10.1002/clc.23925 (PMC9748756; doi:10.1002/clc.23925)

**Supplementary Figure 1: Study flowchart**

COPD, chronic obstructive pulmonary disease; NT-proBNP, N-terminal pro-B-type natriuretic peptide.


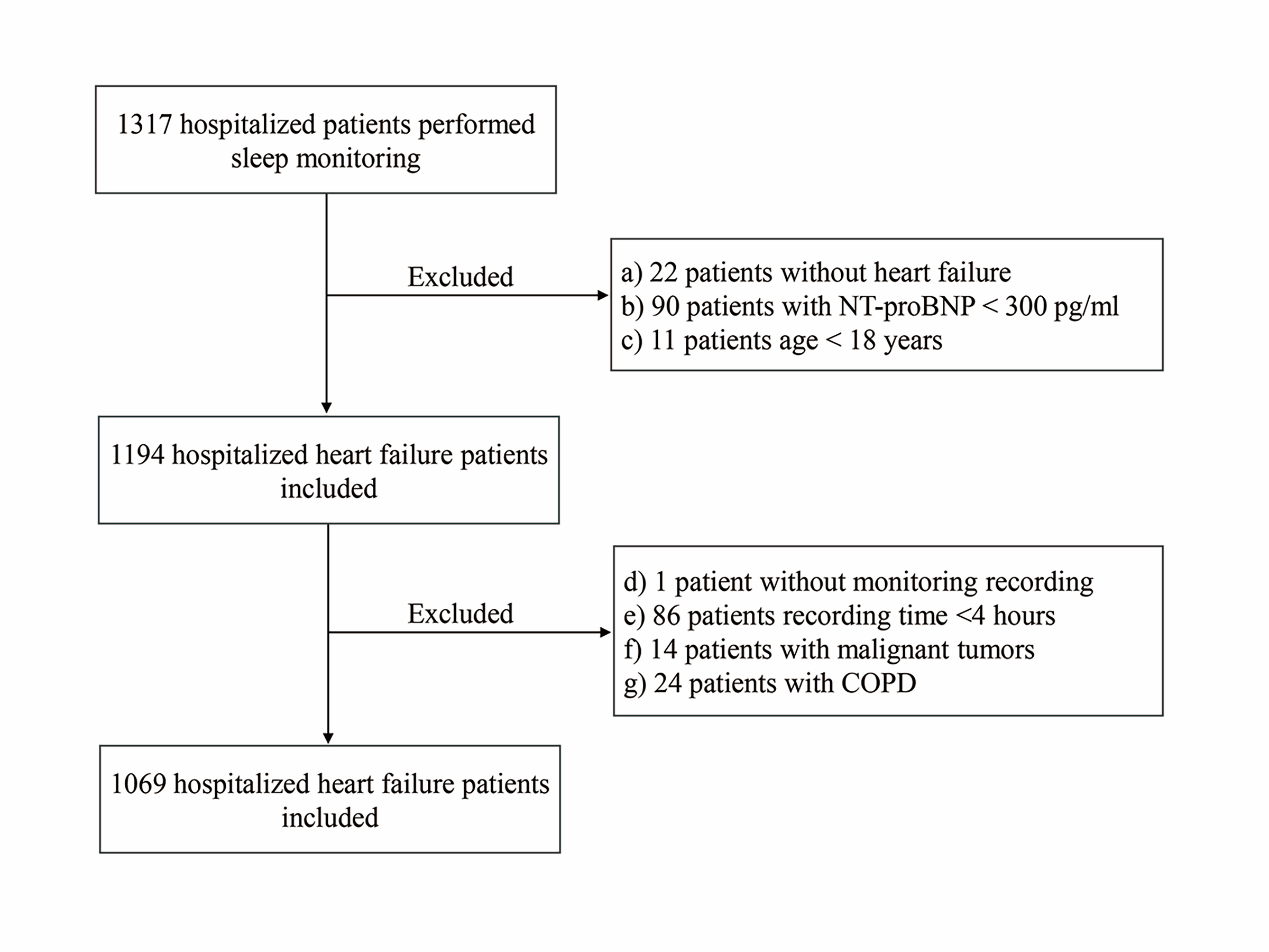

Supplement: Supplementary file 1 — Supporting information. [file CLC-45-1311-s001.docx]
